# Serum Sialylation Changes in Actinic Keratosis and Cutaneous Squamous Cell Carcinoma Patients

**DOI:** 10.3390/jpm11101027

**Published:** 2021-10-15

**Authors:** Mircea Tampa, Ilinca Nicolae, Cristina Iulia Mitran, Madalina Irina Mitran, Cosmin Ene, Clara Matei, Simona Roxana Georgescu, Corina Daniela Ene

**Affiliations:** 1 Department of Dermatology, ‘Carol Davila’ University of Medicine and Pharmacy, 020021 Bucharest, Romania; tampa_mircea@yahoo.com (M.T.); matei_clara@yahoo.com (C.M.); 2 Department of Dermatology, ‘Victor Babes’ Clinical Hospital for Infectious Diseases, 030303 Bucharest, Romania; 3 Department of Microbiology, ‘Carol Davila’ University of Medicine and Pharmacy, 020021 Bucharest, Romania; cristina.iulia.mitran@gmail.com (C.I.M.); madalina.irina.mitran@gmail.com (M.I.M.); 4 Departments of Urology, ‘Carol Davila’ University of Medicine and Pharmacy, 020021 Bucharest, Romania; cosmin85_ene@yahoo.com; 5 Department of Nephrology, ‘Carol Davila’ Nephrology Hospital, 010731 Bucharest, Romania; koranik85@yahoo.com; 6 Departments of Nephrology, ‘Carol Davila’ University of Medicine and Pharmacy, 020021 Bucharest, Romania

**Keywords:** cSCC, AK, sialylation, sialyltransferase, sialidase

## Abstract

Cutaneous squamous cell carcinoma (cSCC), a malignant proliferation of the cutaneous epithelium, is the second most common skin cancer after basal cell carcinoma (BCC). Unlike BCC, cSCC exhibits a greater aggressiveness and the ability to metastasize to any organ in the body. Chronic inflammation and immunosuppression are important processes linked to the development of cSCC. The tumor can occur de novo or from the histological transformation of preexisting actinic keratoses (AK). Malignant cells exhibit a higher amount of sialic acid in their membranes than normal cells, and changes in the amount, type, or linkage of sialic acid in malignant cell glycoconjugates are related to tumor progression and metastasis. The aim of our study was to investigate the sialyation in patients with cSCC and patients with AK. We have determined the serum levels of total sialic acid (TSA), lipid-bound sialic acid (LSA), beta-galactoside 2,6-sialyltransferase I (ST6GalI), and neuraminidase 3 (NEU3) in 40 patients with cSCC, 28 patients with AK, and 40 healthy subjects. Data analysis indicated a significant increase in serum levels of TSA (*p* < 0.001), LSA (*p* < 0.001), ST6GalI (*p* < 0.001), and NEU3 (*p* < 0.001) in the cSCC group compared to the control group, whereas in patients with AK only the serum level of TSA was significantly higher compared to the control group (*p* < 0.001). When the cSCC and AK groups were compared, significant differences between the serum levels of TSA (*p* < 0.001), LSA (*p* < 0.001), ST6GalI (*p* < 0.001) and NEU3 (*p* < 0.001) were found. The rate of synthesis of sialoglycoconjugates and their rate of enzymatic degradation, expressed by the ST6GalI/NEU3 ratio, is 1.64 times lower in the cSCC group compared to the control group (*p* < 0.01) and 1.53 times lower compared to the AK group (*p* < 0.01). The tumor diameter, depth of invasion, and Ki67 were associated with higher levels of TSA and LSA. These results indicate an aberrant sialylation in cSCC that correlates with tumor aggressiveness.

## 1. Introduction

Cutaneous squamous cell carcinoma (cSCC) together with basal cell carcinoma (BCC) represent the most frequent non-melanoma skin cancers [1]. Unlike BCC, cSCC may exhibit an aggressive behavior with a great ability to metastasize to any organ in the body. The most important risk factors associated with cSCC are sun exposure, fair skin phototype, age (mainly diagnosed in middle-aged and older adults), certain beta human papillomavirus (HPV) types [2], and immunosuppression [2,3]. It is well known that cSCC arises on damaged skin, on sites characterized by chronic inflammation such as scars or burns [4]. Sun-exposed keratinocytes produce a wide range of molecules (e.g., inflammatory cytokines, chemokines, growth factors, etc.) that induce increased vascular permeability and the recruitment of immune cells such as neutrophils, macrophages, etc. However, UV radiation leads to the depletion of Langerhans cells in the epidermis [5]. UV radiation promotes the formation of an inflammatory milieu that contributes to skin carcinogenesis [6]. Chronic inflammation is a cofactor for tumor development and induces local immunosuppression facilitating tumor invasiveness and metastasis. Immunocompromised individuals have a 65- to 250-fold increased risk of developing a cSCC [4,7]. Regarding HPV infection, several studies suggest the role of beta HPV types in the pathogenesis of cSCC [4,8,9,10]. Beta HPV DNA has been identified in cSCC samples and antibodies against HPV have been detected in the serum of cSCC patients [4]. The main factors predicting a poor outcome are depth of invasion higher than 2 mm, a low degree of differentiation, location in high-risk areas (face, ear, hands, feet, genitalia), perineural involvement, and the presence of multiple tumors [11,12,13]. cSCC can occur de novo or from the histological transformation of preexisting actinic keratoses (AK), which represent the intradermal proliferation of dysplastic keratinocytes and display potential for malignant transformation into non-melanoma skin cancer, giving rise especially to SCC [14,15].

Carcinogenesis is frequently associated with abnormal sialylation of glycoproteins and glycolipids as a consequence of changes in the activity of sialyltransferases and sialidases [16,17,18,19]. Sialic acids are negatively charged sugars that commonly are coupled to the terminal carbohydrate chains of glycoproteins and glycolipids [20]. Aberrant expression of sialic acid plays a crucial role in tumor aggressiveness by promoting cell proliferation, cell-cell interaction, cell migration, angiogenesis, and tumor metastasis [17,19,21,22,23]. Total serum sialic acid (TSA) and lipid-bound sialic acid (LSA) are significantly elevated in skin cancers [18,24]. Hypersilalylation influences immune cell responses. Sialic acid-binding receptors such as Siglecs modulate the activity of immune cells in the tumor microenvironment, leading to an abnormal inflammatory response [25]. Siglecs are involved in tumor progression and immune evasion, for example, engagement of Siglec-9 or Siglec-E on neutrophils prevents neutrophil-mediated killing of malignant cells [26].

Sialylation is a process mainly governed by sialyltransferases and sialidases [27]. The transfer of sialic acids is modulated by sialyltransferases, a group of glycosyltransferases divided into four families: β-galactoside α2,3-sialyltransferases (ST3Gal-I-VI), β-galactoside α2,6-sialyltransferases (ST6Gal-I and -II), GalNAc α2,6-sialyltransferases (ST6GalNAc-I-VI), and α2,8-sialyltransferases (ST8Sia-I-VI) [28]. During cell differentiation and neoplastic transformation, the expression of sialyltransferases undergoes substantial alterations resulting in phenotypic changes [27,28,29,30]. Sialyltransferases are well known as crucial modulators of several important processes such as cell-cell communication, cell-matrix interaction, cell adhesion, cell signaling, and trafficking [29]. Sialidases, or neuraminidases, are glycohydrolytic enzymes that catalyze the hydrolysis of α−glycosidically linked sialic acid residues from carbohydrate groups of glycoproteins and glycolipids [31]. To date, four types of human sialidases have been identified: NEU1, NEU2, NEU3, and NEU4 [32]. In cancer, the alteration of sialidase activity was associated with cell proliferation, invasion, and metastasis [33]. 

We have previously investigated sialoglycoconjugate abnormalities and anti-ganglioside immune response as possible mechanisms involved in oncogenesis (cutaneous melanoma [23], clear cell renal cell carcinoma [34]), autoimmune diseases (systemic lupus erythematosus, lupus nephritis [35]), and diabetes [36]. Increased sialylation in melanoma cells could represent an event associated with the progression of cutaneous melanoma [17]. Sialoglycoconjugates may promote processes that are involved in the modulation of host immune and inflammatory responses [37]. Sialylation, manifested as the overexpression of TSA, LSA, and orosomucoid, has been shown to be an early, well-expressed event in the initial stages of clear cell renal cell carcinoma [34]. In the medical literature, there are few studies that have analyzed sialylation in cSCC and AK [29,38,39,40]. Therefore, the data are scarce and inconclusive. The aim of our study is to investigate the sialyation in patients with cSCC and patients with AK and find reliable serum parameters useful in the diagnosis of cSCC. To achieve these goals, we have investigated both the levels of sialic acid (TSA and LSA) and the levels of the enzymes involved in its metabolism (ST6GalI and NEU3). In addition, we have analyzed the relationship between the studied parameters and the histological characteristics of the tumor (diameter, depth of invasion, Ki67, and ulceration).

## 2. Materials and Methods

### 2.1. Study Participants

We have conducted a study on 40 consecutive patients with cSCC, 28 consecutive patients with AK and 40 healthy subjects as controls, with skin phototypes I–IV. The patients and controls were matched by age and sex. In the control group, we included healthy subjects who addressed the dermatology clinic for disorders such as skin tags or nevi, disorders that do not interfere with our determinations. In all cases, the diagnosis was confirmed histopathologically. Informed consent was obtained from all study participants. The procedures and experiments were conducted according to the Declaration of Helsinki. The study protocol was approved by the Ethics Committee of the Victor Babes Infectious and Tropical Diseases Hospital (3/10.03.2018).

### 2.2. Histopathological Examination

**Figure 1:**
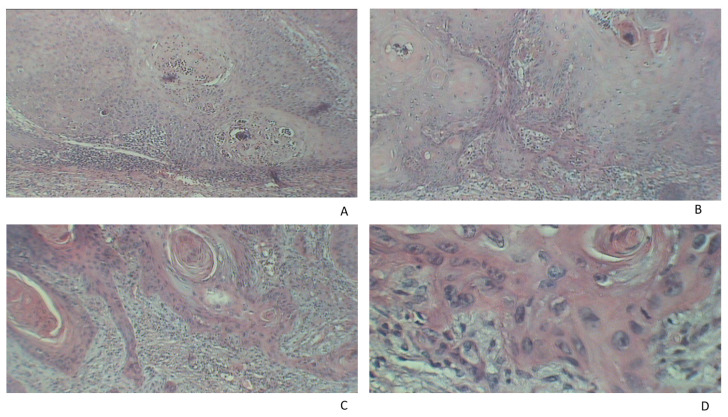
Histopathological examination of cSCC. (**A**) Cell proliferation with giant nuclei and nuclear polymorphism (HE, ×40). (**B**) Proliferation of squamous cells which extend into the deeper layers of the skin, abundant inflammatory infiltrate (HE, ×40). (**C**) Massive inflammatory infiltrate distributed throughout the tumor mass and disseminated tumor cells with giant nuclei (HE, ×40). (**D**) Proliferation of squamous cells with nuclear pleomorphism and nuclear abnormalities and abundant inflammatory infiltrate (HE, ×90).

**Figure 2:**
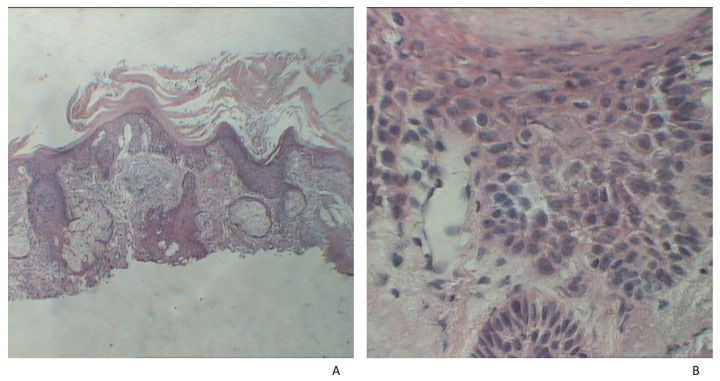
Histopathological examination of AK. (**A**) Disorganized growth, which disrupts differentiation (HE, ×10). (**B**) Dysplasia of basal keratinocytes (HE, ×90).

The tissue samples were fixed in 10% formalin and the method of paraffin embedding was used. For microscopic examination, hematoxylin–eosin stain (HE) was performed (Figure 1 and Figure 2). We have determined the tumor diameter, depth of invasion, and the presence of ulceration. Depth of invasion was calculated as the perpendicular distance between the basement membrane and the deepest point of the infiltrative zone of the tumor according to the recommendations from the 8^th^ Edition of the AJCC [41]. The values were expressed as millimeters.

The Ki-67 antigen is a marker for cell proliferation and is widely used as a cancer activity marker [42]. We used the VECTASTAIN ABC KIT (PK-6101), specifically designed for immunohistochemical staining of tissues. The kit contains biotinylated IgG that was used to bind to the primary anti-Ki67 antibody. The cSCC samples were divided into three subgroups according to the Ki67 index as follows: low < 25%, intermediate 25–75%, and high > 75%.

### 2.3. Laboratory Determinations

Blood samples were drawn from the patients and controls enrolled in the study, under basal conditions using a holder-vacutainer system. The blood samples were centrifuged at 3000× *g* for ten minutes and the supernatant was frozen at −80 °C. The hemolyzed or lactescent samples were rejected.

The serum levels of sialic acid were determined using resorcinol-chlorohydric acid. Blue chromophore was extracted with n-butyl/n-butanol acetate and the optical density was measured at 580 nm with the Sigma reactive (SIALICQ Kit) and the BS3000 analyzer (SINNOWA Medical Science and Technology, Nanjing, China). The results were expressed as mg/dL.

The determination of lipid-bound sialic acid (LSA) levels was performed as follows: 50 µL serum was diluted with 150 µL of cold distilled water. There was added 3 mL of chloroform:methanol (*v*/*v*) 2:1, at 4 °C. The extraction and partition were made after adding 0.5 mL of cold distilled water. After separating the phases by centrifugation, sialic acid was titred with resorcinol-chlorhidric acid.

The serum levels of beta-galactoside 2,6-sialyltransferase I (ST6GalI) (E.C.2.4.99.1) were assessed by ELISA method-sandwich variant (IBL. Co., Ltd., 27762 kit, Okayama, Japan) using a Tecan analyzer (Männedorf, Switzerland). The method is sensible (0.20 ng/mL), reproducible (95–97%), both intra-assay coefficient of variation (CV) and inter-assay CV are less than 15%. It has large limits of detection (1.09–70 ng/mL). The technique uses two kinds of highly specific antibodies. The colorimetric evaluation of the final product was made at a wavelength of 450 nm. The concentration of ST6GalI in the samples was determined by comparing the optical density of the samples to the standard curve. The results were expressed as ng/mL serum.

The serum levels of human sialidase-3 (NEU3) (E.C.3.2.1.18) were assessed by ELISA method-sandwich variant (Mybiosource. Cat. No:MBS 9368355) using a Tecan analyzer (Männedorf, Switzerland). The method is sensible (0.1ng/mL), reproducible (95–97%), both intra-assay CV and inter-assay CV are less than 15%. It has large limits of detection (0.25 ng/mL–8 ng/mL). The colorimetric evaluation of the final product was made at a wavelength of 450 nm. The concentration of NEU3 in the samples was determined by comparing the optical density of the samples to the standard curve. The results were expressed as ng/mL serum.

### 2.4. Statistical Analysis

Triple comparison of the groups was performed using Kruskal–Wallis test and the Dunn post hoc test and pairwise comparison of the groups was performed using Mann–Whitney U test, according to the data distribution (evaluated by Kolmogorov–Smirnov test). The relationship between pairs of two parameters was assessed by Spearman’s correlation coefficient, according to the data distribution. We chose a significance level (*p*) of 0.05 (5%) and a confidence interval of 95% for hypothesis testing.

## 3. Results

Descriptive data of the study participants and the characteristics of the tumors are presented in Table 1.

**Figure 3:**
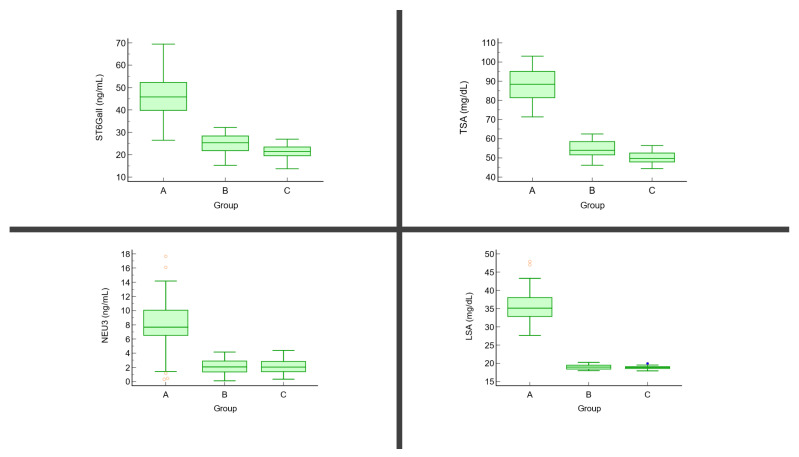
The serum levels of TSA, LSA, ST6GalI, and NEU3 in the studied groups (A = cSCC group, B = AK group, C = control group).

The serum levels of TSA and LSA were higher in the cSCC group compared to the controls. In the AK group, only the TSA levels were higher compared to the control group. In addition, there were significant differences between the cSCC and AK groups. The serum levels of ST6GalI and NEU3 were higher in the cSCC group and AK group compared to the controls, the differences were statistically significant only when we compared cSCC patients to the control group. In addition, there were significant differences between the cSCC and AK groups (Table 2, Figure 3).

The rate of synthesis of sialoglycoconjugates and their rate of enzymatic degradation, expressed by the ST6GalI/NEU3 ratio, is 1.64 times lower in the cSCC group compared to the control group (*p* < 0.01) and 1.53 times lower compared to the AK group (*p* < 0.01).

There were statistically significant positive correlations between the serum levels of TSA and LSA and the tumor characteristics (diameter, depth of invasion, and Ki67 index) (Table 3). There were also statistically significant positive correlations between the serum levels of ST6GalI and the tumor characteristics (diameter and Ki67 index) (Table 3).

## 4. Discussion

The pathogenesis of cSCC is complex and includes numerous intrinsic and extrinsic factors [43,44,45]. Carcinogenesis is a multistep process involving many cells, mediators, and signaling pathways [46,47]. In carcinogenesis, significant structural changes have been described regarding the carbohydrates in the structure of glycoproteins and glycolipids such as a high number of polylactosaminnoglycan chains, multiple branching of asparagine-linked glycans, and increased sialylation [33]. Sialic acid is the outermost monosaccharide unit in the glycan chains of glycolipids and glycoproteins [48] and is ubiquitously distributed in the human body being involved in both physiological and pathological processes. Sialic acid participates in physiological processes such as the transport of positively charged compounds or conformational changes of glycoproteins on cell membranes and mediates cell–cell interactions [21,49]. Elevated levels of sialic acid are associated with resistance to apoptosis and altered cell interactions, promoting cell survival and migration [50]. Abnormal activity of the enzymes involved in sialic acid metabolism or abnormal expression of their corresponding genes leads to aberrant sialylation of glycoproteins or glycolipids [51]. Malignant cell surface glycoproteins and glycolipids display a modified sialic acid composition [39]. Changes in the structure of these glycoproteins and glycolipids may impair processes such as cell recognition, adhesion, or antigenicity. Thus, nowadays sialylation is regarded as one of the major features of the malignant process [21,49]. Aberrant sialylation is the main cancer-associated change of glycosylation process [52,53]. Therefore, it has been suggested that the serum levels of sialic acid and enzymes of sialic acid metabolism may be useful markers in monitoring cancer patients [49]. Parmar et al. have shown higher serum levels of sialic acid in patients with head and neck SCC compared to the control group [38]. Vural et al. have detected increased serum TSA levels in patients with actinic keratoses compared to controls. When they compared the serum TSA levels between AK patients and BCC patients, no differences were obtained [39]. In the current study, we have also detected higher serum levels of TSA in the patient groups compared to controls. However, the serum levels of LSA were significantly higher only when we compared cSCC patients to the control group. In cSCC patients, we have identified higher serum levels of both TSA and LSA compared to AK patients. These results indicate that biochemical changes in glycoproteins start at an early stage of tumorigenesis. High serum levels of LSA are indicative of a premalignant change. Krishnan et al. have shown a positive correlation between TSA level and the grade of dysplasia in patients with oral leukoplakia, but no specific changes regarding LSA levels and the grade of epithelial dysplasia [54].

In various types of cancer, alteration in sialyltransferase activity has been observed [55,56]. ST6GalI seems to be the major sialyltransferase that is overexpressed in malignant tumors [57]. ST6GALI is upregulated in several cancers such as breast, ovarian, and pancreatic neoplasms, being involved in tumor aggressiveness and metastasis [27,57]. Recent studies have demonstrated the role of ST6GALI in the malignant phenotype (growth, survival, angiogenesis, apoptosis, invasion, resistance to cell stress, chemoresistance). Increased ST6GAL1 activity is determined by genetic instability and epigenetic, transcriptional, and posttranslational factors [27,30].

Although there are several studies that identified overexpression of ST6GalI in advanced disease [58,59], a study evaluating patients with oral SCC (OSCC) has revealed ST6GalI overexpression in the early stages of the disease [60]. In line with this, recently, Mehta et al. have analyzed mRNA expression of a group of sialyltransferases, including ST3GALI, ST3GALII, ST3GALIII, ST3GALIV, ST3GALVI, ST6GALI, and the plasma membrane-associated sialidase NEU3, and found down-regulation of these transcripts in OSCC samples compared to adjacent healthy tissue. In normal tissue, they observed increased mRNA levels of sialyltransferases and sialidase NEU3. These results may indicate that alterations of glycosylation are very early events during carcinogenesis. Probably in fact, in the adjacent tumor tissue, although macroscopically normal, a population of cells with early genetic changes is present. The authors also demonstrated that elevated mRNA levels of ST3GALII and ST3GALIII may be prognostic markers in OSCC [61].

We have detected higher serum levels of ST6GalI in cSCC patients compared to controls, whereas there were no significant differences between AK patients and controls. The soluble form of ST6GalI, possibly secreted by SCC cells, may participate in cancer progression and metastasis. This finding is supported by a previous study that showed high immunohistochemical expression of ST3GalI and ST6GalI in cutaneous epithelial lesions including keratoacanthoma, AK, BCC, and SCC. These results suggest that sialyltransferases are deregulated in skin tumors. No significant differences were found between SCC and AK samples [29]. In our study, we have found higher serum levels of ST6GalI in cSCC compared to AK. These findings show that AK and cSCC have a different pattern of sialylation that is related to tumor behavior. Only a small number of AK suffer malignant transformation [62].

Plzak et al. found that poorly differentiated SCCs are positive for 2,6-linked NeuNAc, whereas differentiated SCCs express 2,3-linked-NeuNAc [63]. The cleavage of 2,6-linked NeuNAc by sialidases allows tumor cells to be recognized by Gal-3, a useful prognostic marker in head and neck SCC. It has been shown that 2,6-NeuNAc is a more potent masking compound than 2,3-NeuNAc with regard to the binding of Gal-3 [63,64]. Melanoma is a very aggressive tumor [65] and a recent study has highlighted the role of ST3GALI in melanoma metastasis [50]. Inhibition of enzyme activity is associated with decreased invasion capacity of melanoma cells and reduced ability to hematogenously disseminate. In this process, the ST3GALI–AXL axis may have a role, where the receptor tyrosine kinase AXL represents the key effector of the ST3GALI pro-invasive function. Modulation of the ST3GAL1–AXL axis may represent a target in melanoma treatment [50]. 

The plasma membrane-associated sialidase NEU3 is upregulated in various types of cancer including renal, colon and prostate neoplasms [66,67,68]. In malignant tumors, NEU3 promotes cell survival, migration, and adhesion [69]. NEU3 induces carcinogenesis primarily by altering cell signaling at the cell surface [22]. Recent data have revealed that NEU3 modulates transmembrane signaling through the interaction with various molecules such as caveolin-1, Rac-1, integrin β4, and epidermal growth factor receptor (EGFR) [33]. In colon neoplasms, NEU3 mediates tumor cell proliferation through integrin-mediated signaling, depending on the extracellular matrix. Therefore, NEU3 produces increased adhesion to laminin, promoting cell proliferation and decreased adhesion to fibronectin [70]. On the other hand, in renal cancer, increased levels of NEU3 mRNA have been identified in association with high levels of IL-6, a cytokine that acts as an activator of NEU3, and in turn, NEU3 drives IL-6-mediated signaling via the PI3K/Akt pathway leading to a malignant phenotype characterized by reduced apoptosis and increased cell mobility [67].

Hata et al. suggested that the serum levels of NEU3 are correlated with serum gangliosides, which have been detected in patients with neoplasms [22]. It seems that NEU3 is released from the cell surface in response to the accumulation of gangliosides in serum. It should be noted that NEU3 is an important enzyme for ganglioside degradation [22]. NEU3 acts on gangliosides that are located on the same cell membrane (cis-activity) or on the membrane of neighboring cells (trans-activity), regulating the interactions between cells [71]. The overexpression of *NEU3* and *GD3* synthase genes has been reported in cutaneous melanoma. NEU3 and GM1 and GM2 synthases, involved in ganglioside metabolism, were associated with melanoma cell proliferation and invasion [72]. NEU3 regulates transmembrane signaling through the modulation of ganglioside catabolism. NEU3 hydrolyzes polysialic acid-containing gangliosides to GM1 ganglioside and is involved in the increase in GM1 levels [73]. In addition, in some instances, gangliosides are not efficiently hydrolyzed by plasma membrane sialidases and this explains why gangliosides may accumulate [72].

In our study, we have found higher serum levels of NEU3 in cSCC patients when compared to the control group and as well as when compared to AK patients. The results were similar between AK patients and the control group. The study by Shiga et al. has revealed that mRNA levels of NEU3 are upregulated in head and neck SCC with lymph nodes metastases compared to normal cells. In addition, the study has shown that NEU3 stimulates cell mobility and invasion. NEU3 increases the phosphorylation of EGFR, which may lead to ERK activation and subsequent release of metalloproteinases (MMP-2 and MMP-9) that promote cell invasion [40]. It has also been shown that NEU3 modulates the phosphorylation of EGFR and its dimerization in HeLa cells, activating the Ras cascade, which will promote cell survival [69].

The results of the present study show that there is an altered degradation rate of sialoglycoconjugates in cSCC patients compared to AK and control groups. Hypersialylation and increased glycoconjugate catabolism are features of patients with cSCC compared to AK patients and controls. Our study has revealed that the ST6GalI/NEU3 ratio is significantly altered in patients with cSCC and could represent a potential molecular target in these patients.

This is necessary to determine markers for SCC diagnosis and management [74]. In our study, the tumor diameter, depth of invasion, and Ki67 were associated with higher levels of TSA and LSA. Inal et al. also found increased levels of LSA that correlated with tumor size in patients with head and neck SCC [75]. There was no correlation between NEU3 and tumor aggressiveness. As mentioned above, NEU3 plays an important role in tumor invasion and metastasis, but metastasis in cSCC is very rare. In line with this, in head and neck SCC, NEU3 has been associated especially with lymph node metastasis [40]

These results support the role of these compounds in tumor progression and invasiveness. A potential role of sialidases as therapeutical targets in the management of cancer has been highlighted. For example, oseltamivir phosphate, an extensively used anti-influenza drug and a viral sialidase inhibitor, targets human NEU1 showing encouraging results in preventing tumor cell metastasis [76,77] The inhibition of NEU1 expression by oseltamivir phosphate leads to overexpression of E-cadherin and downregulation of N-cadherin expression, which may limit the ability of cells to metastasize and increase their sensitivity of antineoplastic drugs [78].

Monitoring serum levels of total sialic acid, lipid-bound sialic acid, sialyltransferases, and sialidases in correlation with tumor characteristics may have useful clinical applications for the diagnosis of cSCC. Future research is required to find reliable markers for the management of cSCC.

## 5. Conclusions

The current study reveals aberrant sialylation in cSCC patients, but not in AK patients. We have found a significant increase in serum levels of TSA, LSA, ST6GalI, and NEU3 in the cSCC group compared to the control group, whereas in patients with AK only the serum level of TSA was significantly higher compared to the control group. When the cSCC and AK groups were compared, significant differences between the serum levels of TSA, LSA, ST6GalI, and NEU3 were found. In conclusion, the serum ST6GalI/NEU3 level may represent a potential molecular factor to distinguish cSCC patients from non-cancer patients, pending further validation. Further studies are needed to understand sialylation-related changes in cSCC, supporting further improvements in the diagnostic and treatment approach.

## Figures and Tables

**Table 1:** Clinical data of the study participants and tumor characteristics.

	cSCC Group (*n* = 40)	AK Group (*n* = 28)	Control Group (*n* = 40)
Patient characteristics			
Female/male	16/24	12/16	18/22
Age (years)	56.6 ± 10.8	55.8 ± 8.5	54.6 ± 9.3
BMI (kg/m^2^)	23.6 ± 1.7	22.4 ± 1.4	22.1 ± 1.9
Skin phototypes I-II/III-IV	16/24	15/13	22/18
Exposure/non-exposure to UV	34/6	24/4	32/8
Tumor characteristics			
Tumor diameter <2 cm/>2 cm	28/12	28/0	-
Depth of invasion <4 mm/>4 mm	23/17	-	-
Ki67 index: <25%/25–75%/>75%	15/14/11	-	-
Lesion ulceration - Present/absent	23/17	1/27	-

cSCC—cutaneous squamous cell carcinoma; AK—actinic keratosis.

**Table 2:** The serum levels of TSA, LSA, ST6GalI, and NEU3 in the studied groups expressed as the mean ± standard deviation.

Parameter	cSCC Group (*n* = 40, A)	AK Group (*n* = 28, B)	Control Group (*n* = 40, C)	*p* *	*p* **
TSA (mg/dL)	86.26 ± 8.58	56.41 ± 4.27	49.71 ± 3.56	*p* < 0.01	A vs. B: <0.001 A vs. C: <0.001 B vs. C: <0.001
LSA (mg/dL)	36.59 ± 7.14	18.92 ± 0.56	18.77 ± 0.49	*p* < 0.01	A vs. B: <0.001 A vs. C: <0.001 B vs. C: 0.79
ST6GalI (ng/mL)	49.06 ± 10.02	24.62 ± 3.71	22.31 ± 2.90	*p* < 0.01	A vs. B: <0.001 A vs. C: <0.001 B vs. C: 0.052
NEU3 (ng/mL)	7.64 ± 4.22	2.32 ± 1.16	2.27 ± 1.01	*p* < 0.01	A vs. B: <0.001 A vs. C: <0.001 B vs. C: 0.89

cSCC—cutaneous squamous cell carcinoma; AK—actinic keratosis; TSA—total sialic acid; LSA—lipid-bound sialic acid; ST6GalI—beta-galactoside 2,6-sialyltransferase I; NEU3—neuraminidase 3; *p*-significance level, *p* *—triple comparison of the groups, *p* **—pairwise comparison of the groups.

**Table 3:** Correlations between the serum levels of TSA, LSA, ST6GalI, and NEU3 and histological features of cSCC.

Parameter	TSA		LSA		ST6GalI		NEU3	
	rho	*p*	rho	*p*	rho	*p*	rho	*p*
Diameter	0.63	<0.001 *	0.58	<0.010 *	0.78	0.01 *	0.58	0.07
Depth of invasion	0.39	0.02 *	0.46	0.01 *	0.29	0.13	0.46	0.11
Ki67	0.34	0.04 *	0.66	0.01 *	0.25	0.82	0.26	0.102
Ulceration	0.12	0.29	−0.02	0.78	0.33	0.42	0.47	0.03 *

cSCC—cutaneous squamous cell carcinoma; TSA—total sialic acid; LSA—lipid-bound sialic acid; ST6GalI—beta-galactoside 2,6-sialyltransferase I; NEU3—neuraminidase 3; Ki67—proliferative index; rho—correlation coefficient; *p*—significance level. *—statistically significant.

## Data Availability

The data presented in this study are available on request from the corresponding author.
